# Honey–Propolis-Enriched Pectin Films for Active Packaging of Soluble Coffee and Matcha Powders

**DOI:** 10.3390/gels11100800

**Published:** 2025-10-05

**Authors:** Daniela Pauliuc, Florina Dranca, Mariana Spinei, Sorina Ropciuc, Mircea Oroian

**Affiliations:** 1 Faculty of Food Engineering, Stefan Cel Mare University of Suceava, 13 Universitatii Street, 720229 Suceava, Romania; daniela.pauliuc@fia.usv.ro (D.P.); sorina.ropciuc@fia.usv.ro (S.R.); m.oroian@fia.usv.ro (M.O.); 2 Integrated Center for Research, Development and Innovation in Advanced Materials, Nanotechnologies, and Distributed Systems for Fabrication and Control (MANSiD), Stefan Cel Mare University of Suceava, 13 Universitatii Street, 720229 Suceava, Romania

**Keywords:** edible films, pectin, honey, propolis, active packaging, powdered beverages

## Abstract

This study reports the development and characterization of novel active edible films based on apple pectin and honey (80:20, *w*/*w*), incorporating raw propolis powder at 0.1%, 0.2%, and 0.3% (*w*/*w*, relative to honey) as a natural source of bioactive compounds for sustainable packaging of soluble coffee and matcha powders. The study aims to provide sustainable and functional packaging solutions capable of maintaining the stability and quality of these powdered beverages. The effects of honey and propolis incorporation on the physicochemical, mechanical, optical, and microbiological properties of the films were systematically evaluated. Propolis addition resulted in decreased tensile strength, elastic modulus, and elongation at break, but did not significantly alter the thermal stability of the films, as evidenced by differential scanning calorimetry and thermogravimetric analysis. Increasing propolis concentrations led to higher total phenolic content and significantly improved antioxidant activity, with the 0.3% formulation exhibiting the most pronounced effect. Application tests demonstrated that the honey–propolis-enriched pectin films effectively preserved the sensory attributes and physicochemical quality of soluble coffee and matcha powders. Overall, these results highlight the potential of pectin–honey–propolis films as bioactive carriers and functional materials for active packaging of powdered beverages.

## 1. Introduction

The development of biodegradable materials and edible films and coatings for food packaging that are both environmentally friendly and safe for human consumption is necessary [1]. In recent years, consumers are showing increased interest in packaging materials with potential to extend shelf life and enhance the quality of food products. This growing interest, along with the demand for biodegradable packaging materials, is largely driven by the need to reduce resource waste and mitigate the environmental impact of synthetic packaging materials, which has emerged as a critical global challenge [2]. As a result, the production and development of edible films and coatings have gained substantial momentum in both academic and industrial contexts. This is evidenced by a five-fold increase in scientific publications on the subject since 2012 [3]. The upward trend is also reflected in market projections, with the edible films and coatings sector valued at USD 2.70 billion in 2021 and expected to grow at a compound annual growth rate (CAGR) of 7.7% between 2022 and 2028 [1].

Edible films are thin (usually with a thickness <0.3 mm) and flexible sheets produced from materials suitable for use as food packaging. These films are biodegradable, environmentally friendly, safe for human consumption, resistant to ultraviolet radiation, and soluble in both water and fats [4,5]. Initially, edible films and coatings were primarily formulated using single-component systems, typically based on a single type of biopolymer. However, recent advancements in research have increasingly emphasized the development of multi-component edible materials, which demonstrate enhanced functional properties. To formulate edible films, the components mainly used are polysaccharides, proteins, lipids, or composite biopolymeric systems. Polysaccharide- and protein-based films exhibit good barrier properties against gases and oils; however, they generally have poor water vapor resistance due to their hydrophilic nature. In contrast, lipid-based edible films provide superior water vapor barrier properties but lack mechanical strength and structural integrity [5,6]. In multi-component edible materials, composite films or coatings are formulated by combining two or more film-forming agents to achieve superior mechanical strength, barrier properties, and stability compared to those produced from individual components. To optimize the performance of these materials, a variety of additives such as plasticizers (e.g., glycerol, polyethylene glycol, propylene glycol, sorbitol), cross-linking agents, emulsifiers, and functional enhancers (e.g., vegetable fatty acids) are incorporated into film-forming formulations [4]. Nevertheless, excessive inclusion of certain additives, such as polypropylene glycol or sorbitol, can adversely affect mechanical strength and increase water vapor permeability.

Among polysaccharides with film-forming potential, pectin is regarded as a promising polymer matrix for the development of edible films, owing to its excellent gelling capacity, edibility, biodegradability, and biocompatibility [7]. The gel-forming ability of pectin facilitates the production of thin and flexible film structures. However, films composed solely of pectin typically exhibit poor moisture barrier and limited mechanical and physical properties, which restrict their practical applications in food packaging. Consequently, recent research on the film-forming properties of pectin has focused not only on the influence of pectin source, including that derived from industrial by-products, but also on its modification through blending with other materials, such as complementary polymers, plasticizers, and essential oils, to tailor the structural and functional properties of the resulting films. Research to date has evaluated the formulation of edible films based on blends of pectin with other biopolymers such as chitosan [8], pullulan [9], alginate [10], and cellulose [7,11]. Additionally, studies have investigated the effects of various plasticizers, including glycerol, sorbitol, and polyethylene glycol, on the physical, mechanical, and barrier properties of pectin-based edible films and coatings [10,12,13,14].

Incorporation of antioxidants into pectin-based films has been shown to enhance both the functional and sensory characteristics of the resulting packaging materials [1]. Although only a limited number of bee products have been employed to improve the performance of edible films, some studies have demonstrated their potential. Honey plays a multifunctional role in edible films, acting as a natural plasticizer due to its sugar content (fructose, glucose, and sucrose), and as a surfactant that enhances surface adhesion and surface affinity. In addition to providing mechanical properties comparable to glycerol-based films, honey contributes valuable bioactive compounds that impart antioxidant and antimicrobial functionality, making it an effective component in active food packaging systems [5]. Beeswax has been reported to provide greater stability in film formation compared to conventional agents such as paraffin and octadecanol, and it also enhances the antioxidant and antifungal activities of the films [15,16]. Ethanolic extracts of propolis have been shown to slightly reduce water vapor permeability and exhibit antimicrobial activity against *Listeria innocua* and *Staphylococcus aureus* [17], while the incorporation of bee bread oil has been reported to improve the mechanical properties of pectin films [18].

The present study aimed to investigate the incorporation of honey and propolis, added in raw powdered form, as sources of bioactive compounds in the formulation of edible films based on apple pectin. To the best of our knowledge, this is the first report describing both the development of such a film formulation and its application in soluble beverage products. The physicochemical, microbiological, optical, and mechanical properties, as well as the antioxidant activity, of apple pectin–honey films containing 0.1%, 0.2%, and 0.3% propolis were evaluated, alongside their suitability as packaging for soluble coffee and tea powders.

## 2. Results and Discussion

### 2.1. Film Thickness and Moisture Content

**Figure 1:**
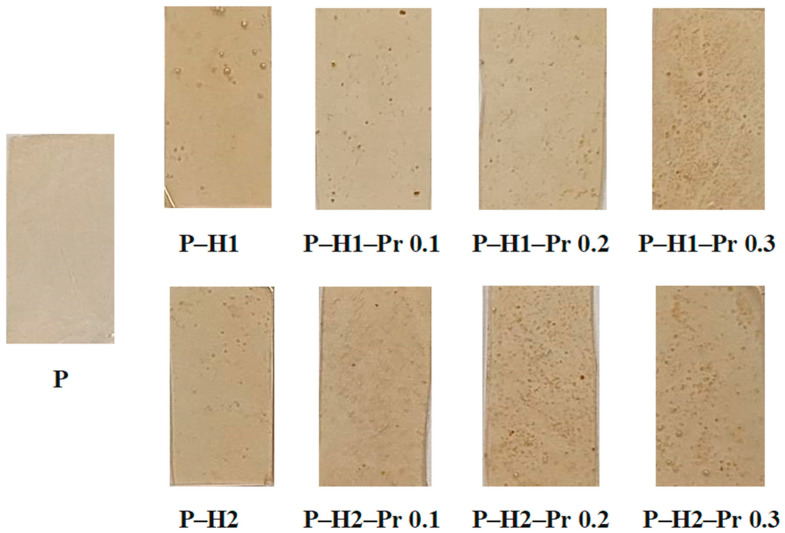
Edible films based on pectin (P), honey (H1 and H2) and propolis (Pr), shown as 30 × 15 mm film sections.

The developed edible films based on pectin and incorporating honey and varying concentrations of propolis (0.1%, 0.2%, and 0.3%), together with the control film containing only pectin, are presented in Figure 1. The visual comparison highlights the uniformity, transparency, and surface characteristics of the films, which represent important preliminary indicators of their structural integrity and potential suitability for application as packaging materials.

The physical properties (thickness and moisture content) of developed films are summarized in Table 1. Clearly, the pectin-based film (P, 70.5 μm) exhibited significantly lower thickness (*p* < 0.05) compared to pectin- and honey-based films incorporated with various concentrations of propolis (0.1, 0.2, and 0.3%), which ranged between 255.8 and 276.9 μm. Moreover, an increase in thickness was observed with higher concentrations of propolis; this enhancement can be attributed to the total solids of blended components, which contribute to a more expanded pectin–honey–propolis network during film formation [17]. A similar tendency was observed for coffee pulp pectin-based films incorporated with propolis extract and honey [5] and for apple pectin-based films with *Apis mellifera* honey and different concentrations (0, 2.5, and 5%) of propolis extract [17], although the films obtained in these studies were thinner when compared to those reported in the present study. Therefore, thickness of the films depends on film forming materials (e.g., polysaccharides), water solubility of blended components, and chemical interaction between forming components [18].

Moisture content (MC) reflects the ability of film matrix to retain moisture after drying process [19]. Therefore, determining both the amount and behavior of water within the film matrix is essential for understanding its physical characteristics [20]. As seen from Table 1, MC of the developed films ranged from 10.05 to 17.57%. The pectin-based film exhibited significantly lower MC in comparison with all other formulations (*p* < 0.05); this increase can be associated with the hydrophilic nature of blended components, which enhance water retention in the film matrix [17]. Osuna et al. [17] reported the same tendency and obtained MC values between 10.27 and 13.74% for apple pectin-based films with *Apis mellifera* honey and different concentrations of propolis extract, while Biratu et al. [5] determined moisture content values ranging from 11.85 to 15.80% for coffee pectin-based films with honey.

### 2.2. Optical Properties of the Films

Color is one of the critical quality properties of the films, especially those intended for food packaging or direct consumption [20]. Accordingly, optical parameters, including lightness (L*), chromaticity characteristics (a*, b*, C*_ab_, and h*_ab_), and total color difference (ΔE*) of the developed films were measured and are shown in Table 1; it can be observed that L* values decreased with increasing propolis concentration from 0.1% to 0.3% in pectin and honey-based film formulations (*p* < 0.05). Therefore, the addition of different concentrations of propolis affected the visual appearance, while control sample (pectin-based film, P) is translucent and shows a light amber coloration. The a* values of the samples ranged from −0.05 to −0.74, indicating that films at higher concentration of the propolis (0.3%) show more pronounced greenish tints due to the natural pigments in propolis (e.g., quercetin, *p*-coumaric acid, kaempferol, and ferulic acid) [17]. The other chromatic parameters (b*, C*_ab_, and h*_ab_) presented the same tendency, indicating an increase in yellowish and greenish hues in the formulated films, placing their color within the yellow-green region of the CIE L*a*b* scale. The ΔE* is a useful characteristic to evaluate the capacity of human eye to detect color differences [21]. The ΔE* values enhanced with the propolis concentration, ranging from 4.93 to 6.81 and 6.43 to 10.52 for samples formulated with H1 and H2, respectively. According to the results, differences in color among samples are easily distinguishable for ΔE* values below 5, and become increasingly pronounced for values above 5. Similar results were reported for apple pectin-based films with *Apis mellifera* honey and different concentrations (0, 2.5, and 5%) of propolis extract [17], for citrus pectin-based films with the addition of propolis extract, Fe^3+^, and zein nanoparticles [22], and for coffee pulp pectin-based films incorporated with propolis extract and honey [5].

The opacity is correlated with the transparency and the change in transparency values might be related to the thickness of the films [19]. The values of opacity, light transmission, and transparency are shown in Table 1; films developed with pectin, honey, and propolis presented opacity values ranged from 1.35 to 3.05 compared to P film (8.76). This can be explained by the fact that honey and propolis both contain polyphenols and sugars, which may act as natural plasticizers. By improving polymer chain mobility and reducing crystallinity, these components enhance the uniformity of the film matrix, thereby reducing light scattering and increasing light transmission [5]. Moreover, these variations in opacity may be due to differences in film thickness and molecular arrangement within the films [5]. A material is considered transparent when it exhibits high light transmission [17]. The increase in the concentration of propolis showed significant changes (*p* < 0.05) in light transmission; the most transparent film was P–H2, with the lowest opacity value (1.35) and the highest light transmission (44.70%). The findings are consistent with those reported for apple pectin-based films with *Apis mellifera* honey and different concentrations (0, 2.5, and 5%) of propolis extract [17] and for active pectin-based films incorporated with green propolis extract [23]. The increased protection of the pectin- and honey-based films against visible light and UV is ascribed to the composition of propolis, primarily owing to the presence of phenolic compounds containing multiple chromophores [17].

### 2.3. Mechanical Properties of Edible Films

The mechanical properties of edible films, which include strength and elasticity, represent fundamental parameters in the characterization of these packaging materials, as they determine the ability of the material to maintain its structural integrity under external mechanical stress [7,24]. The tensile strength (TS), elongation at break (%E), and elastic modulus (EM) of the control film, as well as those of edible films incorporating pectin, honey, and propolis, are presented in Table 1. The control film exhibited the highest elastic modulus and tensile strength, coupled with the lowest elongation at break among all formulations, indicating superior resistance to stress and reduced susceptibility to deformation. The incorporation of honey into edible films at an 80:20 pectin/honey ratio resulted in a reduction in EM from 9.79 MPa to 2.96 MPa (P–H1) and 2.69 MPa (P–H2), and a decrease in TS from 5.81 MPa to 1.18 MPa (P–H1) and 1.22 MPa (P–H2). In contrast, %E increased from 23.60% to 34.49% (P–H1) and 33.37% (P–H2). These changes can be attributed to the plasticizing effect of honey components, primarily sugars and water, which disrupt intermolecular interactions within the pectin network, thereby reducing polymer chain cohesion and rigidity and consequently lowering TS and EM. At the same time, the increased molecular mobility enhances the flexibility of the films, leading to greater elongation at break. These results are consistent with previous studies reporting the effects of honey incorporation on the mechanical properties of films [17,25]. The incorporation of propolis into pectin–honey edible films significantly influenced the tensile strength, elastic modulus, and elongation at break. All mechanical parameters decreased with increasing propolis concentration, likely due to the formation of structural discontinuities within the edible film, which reduced the fracture resistance of the film matrix. All mechanical parameters decreased with increasing propolis concentration, likely due to the presence of raw propolis particles distributed within the film matrix, which created structural discontinuities and reduced the fracture resistance of the film matrix. Similar effects have been documented in other studies investigating propolis-enriched films [26,27].

### 2.4. FT-IR Spectra

**Figure 2:**
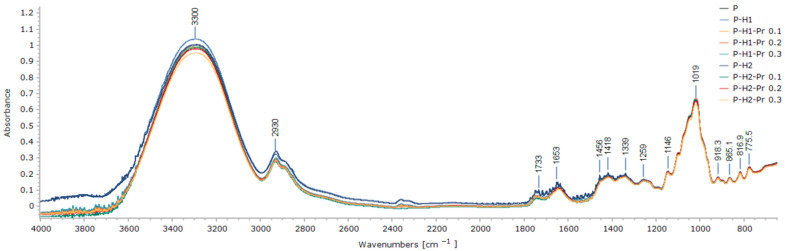
FT-IR spectra edible films based on pectin (P), honey (H1 and H2) and propolis (Pr).

FT-IR spectroscopy was employed to examine the effects of film formulation on the chemical structure of edible films containing pectin, honey, and propolis. The FT-IR spectra of the edible film samples, presented in Figure 2, revealed that the principal characteristic peaks were present in all formulations, irrespective of composition.

The broad absorption band observed at 3300 cm^−1^ was assigned to the stretching vibrations of hydroxyl (O–H) groups within the film matrix, specifically corresponding to the vibrational modes of inter- and intramolecular hydrogen bonds involving the galacturonic acid polymer, as well as to the hydroxyl group vibrations of carbohydrates present in honey [7,28]. Variations in the position or intensity of this band may indicate changes in intermolecular hydrogen bonds [20], reflecting structural modifications within the edible film induced by the incorporation of honey and propolis. Overall, the incorporation of propolis resulted in a decrease in the spectral intensity of the films within the region of 4000–1300 cm^−1^.

The peak at 2930 cm^−1^ was attributed to pectin and corresponded to the stretching vibrations of C–H bonds (CH, CH_2_ and CH_3_ groups), as well as bending vibrations of CH_2_ groups [29]. The peak attributed to C=O stretching in the methylated ester was observed at 1733 cm^−1^, while the peak corresponding to the carboxylic acid group appeared at 1653 cm^−1^ [5]. An increase in the intensity of the esterified carboxyl groups around 1733 cm^−1^, which is indicative of a high degree of pectin esterification, was not observed in the film spectra compared to pure pectin. This is likely due to the overlapping absorption in the 1700–1600 cm^−1^ region, which includes bending vibrations of O–H groups from water, C=O stretching vibrations from fructose, and aldehyde (H–C=O) stretching vibrations from glucose, all characteristic components of honey [28].

Vibrations characteristic of the carbohydrate chemical structure of honey, including stretching vibrations of C–O, C–C, and C–H bonds, as well as bending vibrations of C–H bonds, were assigned to the spectral region between 1470 and 700 cm^−1^ [28]. This region overlaps with the 1200 to 800 cm^−1^ range, which is recognized as the fingerprint region of pectin, where intense peaks are attributed to specific functional groups as well as vibrations associated with glycosidic bonds and pyranose rings (e.g., the peak at 1019 cm^−1^ corresponds to the pyranose structure) [29]. The concentration of propolis incorporated into the edible films was too low to detect the strong absorption peaks previously reported for crude propolis [30].

### 2.5. Thermal Properties of Edible Films

**Figure 3:**
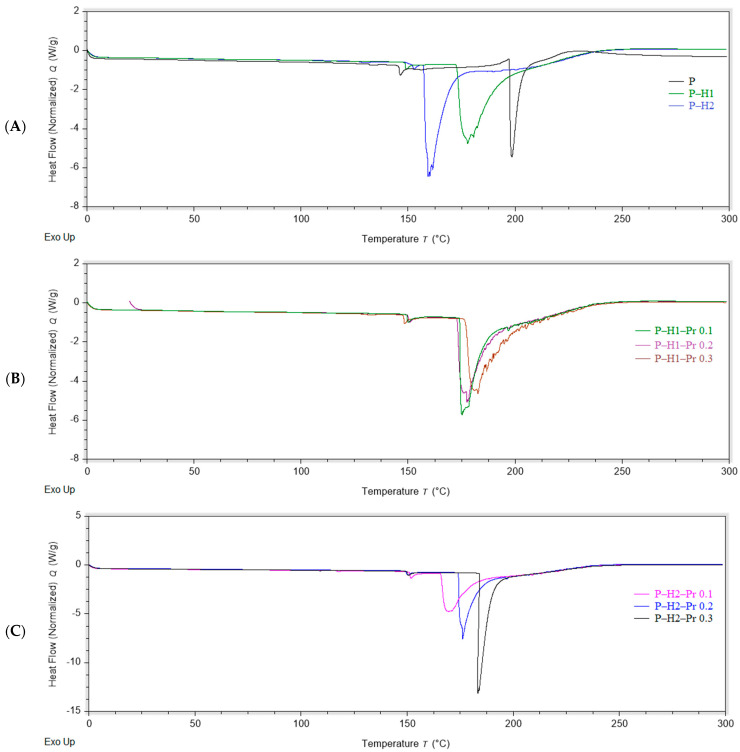
DSC curves showing the effect of propolis incorporation into edible films: (**A**) control film (P) and pectin–honey films (P–H1 and P–H2); (**B**) pectin–honey–propolis films prepared with the first honey sample (H1) and different propolis concentrations; (**C**) pectin–honey–propolis films prepared with the second honey sample (H2) and different propolis concentrations (P—pectin, H—honey, Pr—propolis).

**Figure 4:**
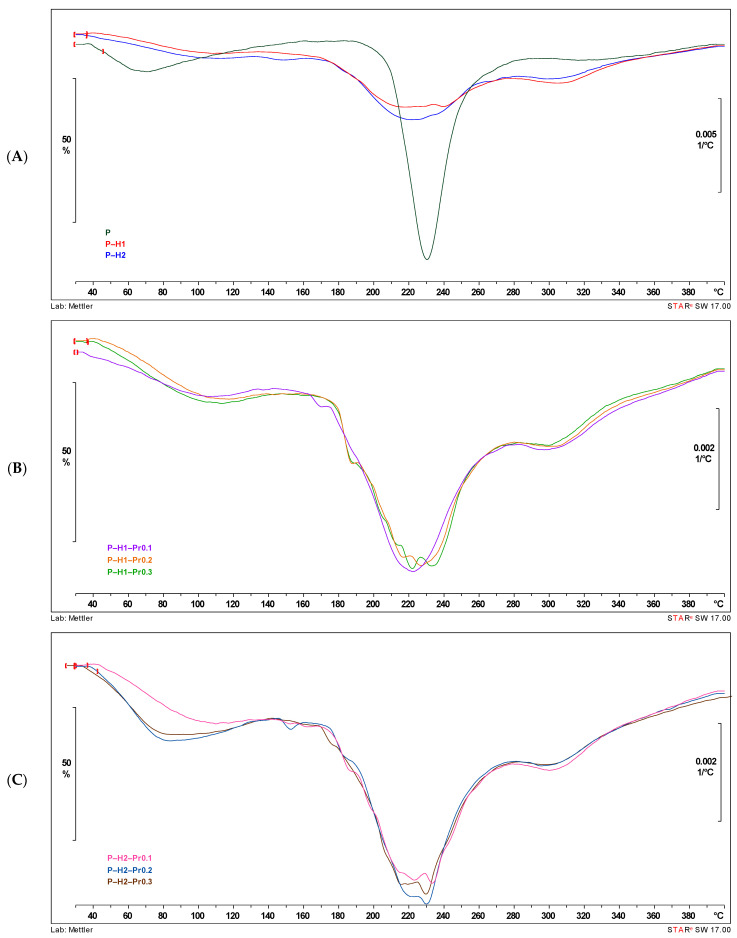
DTG curves showing the influence of honey and propolis incorporation into edible films: (**A**) control film (P) and pectin–honey films (P–H1 and P–H2); (**B**) pectin–honey–propolis films prepared with the first honey sample (H1) and different propolis concentrations; (**C**) pectin–honey–propolis films prepared with the second honey sample (H2) and different propolis concentrations (P—pectin, H—honey, Pr—propolis).

Differential scanning calorimetry (DSC) is commonly employed in the analysis of edible films and other novel packaging materials to assess the compatibility of blend films based on their endothermic transitions [8]. Thermogravimetric analysis (TGA) is a widely applied technique for characterizing polymeric materials, providing information on their thermal stability [31]. The thermal properties of the edible films containing pectin, honey, and propolis are presented in Figure 3 (DSC) and Figure 4 (TGA). The DSC and DTG curves indicate that all edible films displayed similar thermal behavior, characterized by three distinct stages of thermal degradation.

In the DSC curves recorded within the temperature range of 0–300 °C, the edible film samples exhibited two endothermic peaks and one exothermic peak (Table 2). The first endothermic transition was observed at approximately 150 °C, except for formulation P–H2–Pr 0.1, which exhibited this peak at around 137 °C. The second endothermic peak occurred between 159 and 183 °C. Both endothermic events were associated with melting phenomena: the first, smaller peak was likely related to minor conformational changes, whereas the second, sharper peak was indicative of dehydration processes, melting of honey sugars, bond cleavage, and other structural changes preceding degradation of the film matrix, consistent with previous reports [8,32]. The incorporation of honey and propolis significantly (*p* < 0.05) influenced the second endothermic event. In particular, propolis addition resulted in an increase in melting temperature from 177.50 °C (0.1% propolis) to 186.11 °C (0.3% propolis) in one formulation, and from 159.22 °C (0.1% propolis) to 183.34 °C (0.3% propolis) in another. This increase in melting temperature may be attributed to the slower melting of residual substances present in the crude resin characteristic of propolis [33]. Similar effects on the thermal behavior of the matrix have been reported following the incorporation of honey bee brood protein into gel films composed of carboxymethyl starch, chitosan, and pectin [34]. The small exothermic peak observed at 220–260 °C indicated thermal degradation of the edible film, which was consistent with the thermal degradation temperature reported for pectin [29].

Thermogravimetric curves are shown in Figure 4, and the thermal properties of the developed films are presented in Table 3. As shown in Figure 4, the thermal decomposition of the formulated films occurred in three distinct stages: *(1*) the first stage is attributed to the evaporation of residual water and/or volatile compounds, typically occurring between 30 and 150 °C; *(2)* the second stage is attributed to the main thermal decomposition of the pectin polysaccharide chains, sugars from honey (e.g., glucose, fructose), and bioactive compounds in propolis (150–280 °C) [5]; while *(3)* the third stage is correlated with the slow degradation of remaining charred material that occurs between 280 and 350 °C [18]. The highest maximum temperature (T_peak_) during the second stage of thermally induced weight loss, 231.33 °C, was obtained for P–H2–Pr0.2, while the lowest (184.10 °C) was for P–H1–Pr0.3. Although T_peak_ values varied with the addition of propolis, no consistent trend was observed across concentrations or between the H1 and H2 samples. The variation in T_peak_ values may be influenced by several factors, including differences in the dispersion of propolis within the film matrix, the interaction between polyphenolic compounds and the biopolymer network, or slight inconsistencies in thermal behavior due to the complex composition of propolis itself. The residue values at 150 °C, 280 °C, and 400 °C indicate the proportion of material that remains undecomposed at each stage of thermal degradation. Higher residue at 150 °C suggests lower initial moisture and volatile content, while values at 280 °C and 400 °C reflect the thermal stability of the film matrix. Films containing honey (H1, H2) and especially those combined with propolis (Pr0.1–0.3) tend to retain more residue at higher temperatures, indicating enhanced thermal resistance. These differences suggest that honey and propolis modify the film structure and improve its resistance to decomposition. The carbonaceous char residues of the pectin and honey-based films degraded at about 400 °C; at this stage, about 39–52% weight loss was observed for samples enriched with propolis in different concentrations (0.1, 0.2, and 0.3%), while the P–based film showed a weight loss of about 48%. In contrast, Biratu et al. reported a weight loss ranging from 41 to 72%, which differs from the values observed in this study [5]. Therefore, all films demonstrated great thermal stability, indicating their potential to be used as food packaging materials.

### 2.6. Total Phenolic Content, Phenolic Profile, and Antioxidant Activity

The total phenolic content (TPC) is an essential parameter for assessing and understanding the potential therapeutic and biological effects of the developed edible films. TPC of pectin films enriched with honey and propolis is shown in Table 4.

Analysis of the data presented in the table indicates that the highest total phenolic content was recorded in films containing honey supplemented with 0.3% propolis. As shown in Table 4, the films supplemented with 0.3% propolis exhibited the highest values for total phenolic content (TPC) and antioxidant activity (DPPH). The film prepared from pectin, honey, and 0.3% propolis (P–H1–Pr 0.3) displayed a total phenolic content of 54.21 mg GAE/100 g and an antioxidant activity of 59.91%. Similarly, the film P–H2–Pr 0.3, prepared from the second honey sample, pectin, and 0.3% propolis, showed a TPC of 52.90 mg GAE/100 g and an antioxidant activity of 59.89%. A progressive increase in propolis concentration was directly associated with concomitant increases in both total phenolic content and antioxidant activity. These findings highlight the efficacy of propolis incorporation as a strategy for enhancing the functional and bioactive properties of the films. The phenolic profile of the edible films reflects the combined contribution of both honey and propolis. Films composed of pure pectin exhibited low antioxidant activity and a reduced total phenolic content. In their study on pectin films enriched with honey and propolis, Osuna et al. [17] reported that TPC of honey ranged from 83 to 84.6 mg GAE/100 g of sample. The antiradical activity of honey was measured as 14.20% DPPH radical inhibition. Studies on k-carrageenan-based edible films for beef preservation demonstrated that phenolic compounds from honey and bee pollen significantly improved the antioxidant activity [31]. Notably, films containing bee pollen extract exhibited approximately four times higher TPC compared to films with honey extracts, which was attributed to the distinct composition of phenolic acids and flavonoids [31]. Biratu et al. [5] investigated the antioxidant activity of films composed of pectin, pectin–glycerol, pectin–propolis–glycerol, and pectin–honey. They reported that the DPPH inhibition percentage increased in films enriched with honey and propolis compared to films containing only pectin and glycerol. The results indicate that pure pectin and pure honey exhibited lower inhibition values, whereas the incorporation of propolis and honey enhanced the antioxidant capacity of the edible films.

The incorporation of honey and propolis into pectin-based edible films enriched the phenolic compounds profile, thereby enhancing their functional properties. Among the phenolic compounds analyzed, 4-hydroxybenzoic acid was the most abundant, originating from honey; however, its concentration decreased in the films containing propolis compared to those prepared with only pectin and honey (Table 4). In propolis, 4-hydroxybenzoic acid is present only as a minor constituent. Caffeic acid was identified as the second most abundant phenolic compound, followed by myricetin, indicating that the combination of honey and propolis not only increases the total phenolic content but also diversifies the antioxidant profile of the films. These results highlight the potential of honey- and propolis-enriched pectin films as functional materials with applications in food preservation and active packaging. The phenolic compounds identified in the developed edible films were consistent with the phenolic profiles previously reported for honey and propolis [30,35]. Honey is a natural source rich in phenolic acids and flavonoids, including 4-hydroxybenzoic acid, caffeic acid, *p*-coumaric acid, myricetin, quercetin, and kaempferol, which exhibit antioxidant and anti-inflammatory properties [36]. Propolis, similarly, contains high concentrations of flavonoids and phenolic acids, such as caffeic acid, *p*-coumaric acid, myricetin, quercetin, and kaempferol [37]. When combined in a pectin matrix, these compounds are stabilized and protected from oxidative degradation, leading to a significant increase in their measurable levels in the final product. This synergy between honey, propolis, and pectin ensures effective protection of bioactive compounds, maintaining their stability and antioxidant activity under processing and storage conditions [36]. Therefore, utilizing honey and propolis in a pectin matrix is an efficient method to enhance the content of phenolic compounds with antioxidant activity, contributing to the development of functional products with health benefits.

### 2.7. Microbiological Quality of Edible Films

In addition to the intrinsic properties of edible films and coatings, it is of particular importance that such packaging systems are capable of preserving the quality attributes of the packaged food, including the preservation of microbiological parameters in compliance with applicable regulatory standards [38]. Consequently, the microbiological quality of the edible films represents a critical factor and was therefore evaluated in the present study.

The results of the microbiological analysis, including total viable count (TVC), yeast and mold count (YMC), and coliform count (C), for the edible films formulated with pectin, honey, and propolis are presented in Table 5. As indicated, coliform bacteria were not detected in any of the edible film samples, demonstrating the absence of potential fecal contamination and suggesting that the production and handling processes were conducted under appropriate hygienic conditions. Total viable count and yeast and mold count values were generally comparable across formulations, although statistically significant differences were observed between certain samples (*p* < 0.05). For the control films (P), both total viable count and yeast and mold count were lower compared to edible films containing honey and propolis. This difference may be linked to the microbiological characteristics of both propolis and honey; honey, in particular, can contain residual microflora originating from pollen, the digestive tracts of honey bees, dust, air, soil and nectar [39], which may contribute to the observed variability. An increase in propolis concentration was associated with a slight decrease in TVC, which was expected given the well-documented antimicrobial activity of propolis [40,41]. Yeast and mold count values exhibited more pronounced variation (*p* < 0.05) among the different formulations. A marked decrease in YMC was observed with increasing propolis concentration. This observation is consistent with previous reports demonstrating that films enriched with propolis extract significantly inhibit yeast and mold growth; for instance, pullulan films with increasing concentrations of propolis extract displayed strong antifungal and anti-yeast activity, with inhibition occurring in a concentration-dependent manner [26]. Overall, the total viable count and yeast and mold count values determined for the edible films complied with the microbiological limits established by current EU regulations [42], indicating that the films meet safety standards for microbial contamination.

### 2.8. Physicochemical and Sensory Evaluation of Beverages Formulated from Powders Packaged in Edible Films

**Figure 5:**
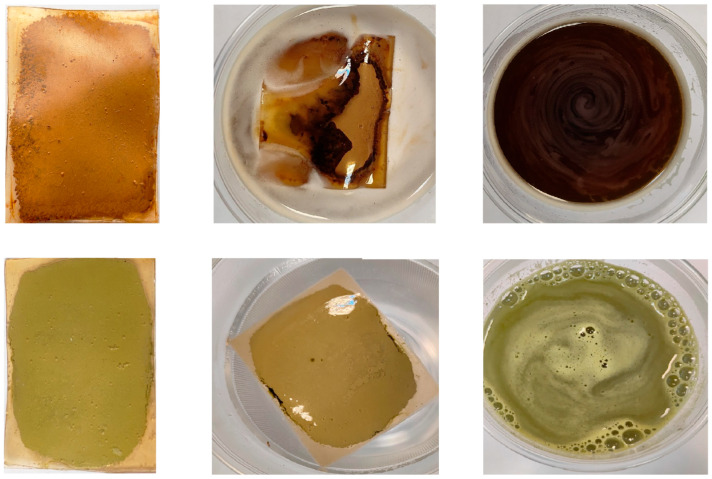
Coffee and matcha powders packed in edible films based on pectin and incorporating honey and propolis and steps of beverages preparation.

The edible films based on pectin, honey, and propolis, characterized in the previous sections with respect to physicochemical properties and microbial quality, were used to package soluble beverage powders, specifically soluble coffee and matcha tea powder. The packaged products measured 50 mm in length, 40 mm in width, and 6 mm in thickness, with a weight of 4.5 g for the coffee packages and 4.0 g for the tea packages (Figure 5). To evaluate the effectiveness of the prepared edible films as packaging for these beverage powders, the beverages were prepared according to the instructions specific to each product: coffee packaged in edible films was immersed in 150 mL of water at 90 °C and stirred until complete dissolution, while tea powder packages were dissolved in 50 mL of water at 80 °C using the same procedure (Figure 5). For all film formulations, complete dissolution of the films was observed within 60 s, except for the control films, which required up to 2 min to fully dissolve.

To evaluate the effect of honey and propolis incorporation into edible films on beverage characteristics and consumer acceptability, beverages prepared from powders packaged in these films were analyzed for color, viscosity, total phenolic content, antioxidant activity (Table 6), and sensory attributes.

Color parameters, including lightness (L*), chromaticity characteristics (a*, b*, C*_ab_, and h*_ab_), and total color difference (ΔE*) of the coffee and matcha beverages are shown in Table 6. The L* values for coffee beverage ranged from 48.18 to 52.54, while those for the matcha beverage varied between 46.12 and 61.01, with statistically significant differences observed among samples (*p* < 0.05). Generally, the addition of propolis increased L* compared to the control sample (coffee beverage—C and matcha beverage—M, respectively). The a* values decreased with propolis concentrations, especially in sample with addition of 0.3% propolis, indicating possible interactions between polyphenols and coffee pigments (e.g., melanoidins and chlorogenic acids). The same tendency was observed for the negative a* values of the matcha samples, confirming the presence of greenish tones characteristic of matcha. The b* values were generally high, reflecting the yellowish chromatic component typical of coffee- and matcha-based beverages. Among the tested edible film formulations, P–H1–Pr0.2 induced the least perceptible color change in the coffee beverage, with a ΔE* value of 0.56. This suggests that it preserves the original appearance of the coffee more effectively than other film types. Similarly, in matcha beverages, the P–H2–Pr0.1 film exhibited the smallest color deviation from the control (ΔE* = 0.82), thereby maintaining the vibrant green tone characteristic of matcha. These results indicate that both films are highly suitable for applications where color integrity is a key quality parameter. Comparable findings were reported for ten commercial matcha green tea samples [43] and for green tea derived from *Camellia sinensis* var Assamica [44]. At the same time, the other chromatic parameters (C*_ab_ and h*_ab_) values indicated that the samples retained relatively consistent color intensity and shade, with observed differences linked to the type of film formulation applied.

As shown in Table 6, the viscosity of the beverages prepared from powders packaged in edible films was influenced by the composition of the packaging material. The reference beverages prepared from powders without edible film exhibited the lowest viscosity values (0.71 × 10^−3^ Pa∙s for coffee and 1.06 × 10^−3^ Pa∙s for matcha), consistent with the absence of any dissolved packaging components. In contrast, packaging with the control edible film containing only pectin resulted in a marked increase in viscosity (1.61 × 10^−3^ Pa∙s for coffee and 2.17 × 10^−3^ Pa∙s for matcha), likely due to the dissolution of pectin macromolecules and their thickening effect, which enhanced the continuous phase viscosity. Beverages prepared from powders packaged in pectin–honey films (P–H1 and P–H2) displayed viscosity values comparable to those of the control pectin films (1.60 × 10^−3^ and 1.59 × 10^−3^ Pa∙s for coffee, 2.13 × 10^−3^ and 2.16 × 10^−3^ Pa∙s for matcha), indicating that at a pectin–honey ratio of 80:20, honey did not significantly modify the rheological behavior. However, the progressive incorporation of propolis (0.1, 0.2, 0.3%) into pectin–honey films caused a gradual decrease in viscosity, which may be attributed to the interference of propolis constituents with the hydration and gel-forming capacity of pectin, thereby reducing the overall thickening effect within the beverage matrix. This decrease in viscosity was consistent with previous studies reporting a reduction in viscosity following the incorporation of propolis into gelatin matrices [45].

Beverages prepared from coffee or matcha powder packaged in pectin-based films exhibited higher values of total phenolic content (TPC) and antioxidant capacity (DPPH) compared with the corresponding reference beverages obtained from unpackaged powders. The increase in TPC and antioxidant activity was positively correlated with the proportion of propolis incorporated into the films. For coffee beverages, TPC values increased from 233.86 mg GAE/100 g in the reference sample to 249.93 mg GAE/100 g in the formulation packaged in edible films containing pectin and honey. In the case of matcha beverages, TPC increased from 282.01 mg GAE/100 g (reference sample) to 298.48 mg GAE/100 g (matcha powder packaged in pectin–honey films). The addition of propolis further enhanced TPC in a concentration-dependent manner, from 273.94 mg GAE/100 g (0.1% propolis film) to 293.21 mg GAE/100 g (0.3% propolis film) for coffee, and from 315.24 mg GAE/100 g to 373.82 mg GAE/100 g for matcha under the same conditions. A comparable trend was observed for antioxidant activity, increasing from 57.08% in the reference matcha beverage to 58.59% in the sample packaged with pectin-based film, and reaching 70.13% when packaged in films containing pectin, honey, and 0.3% propolis. A similar evolution was noted in coffee beverages. These findings demonstrate the concentration-dependent enhancing effect of propolis on both the total phenolic content and antioxidant activity of functional beverages based on coffee and matcha. Jakubczyk et al. [46] reported that the concentration of polyphenolic compounds in tea ranged from 1345.41 to 1765.12 mg/L, with the highest values obtained when brewed at 90 °C for 10 min. For these samples, the antioxidant capacity, expressed as the percentage of DPPH radical inhibition, varied between 12.07% and 41.24%, indicating a moderate to relatively high radical scavenging potential [46]. Similarly, Pintac et al. [47], in their analysis of the phenolic composition and antioxidant activity of commercially available instant coffees and teas, reported that green tea exhibited the highest antioxidant activity per serving, with a TPC of 340 mg GAE/serving, whereas instant coffee provided 187 mg GAE/serving.

**Figure 6:**
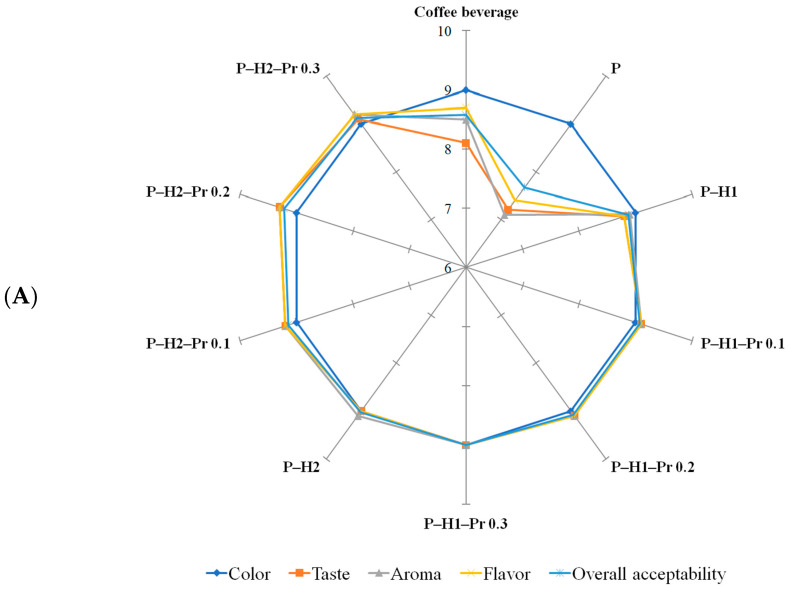

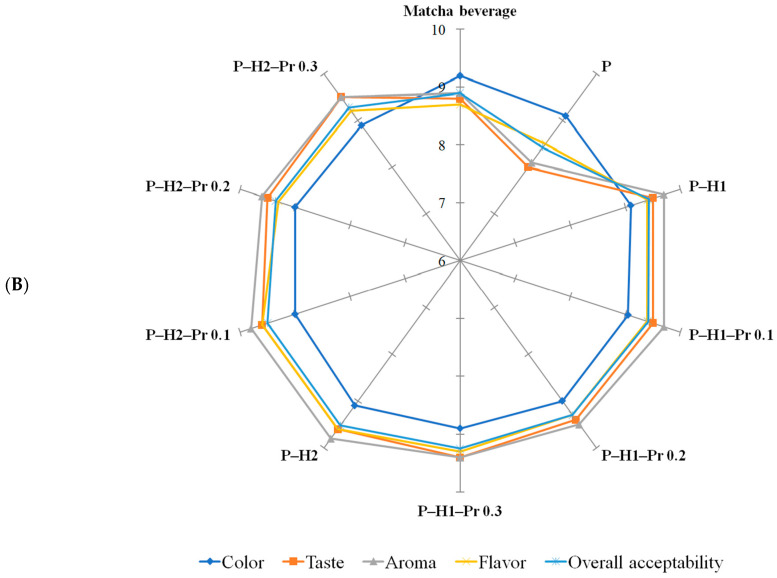
Sensory evaluation of coffee (**A**) and matcha (**B**) beverages formulated from powders packaged in edible films based on pectin (P), honey (H1 and H2) and propolis (Pr).

Sensory evaluation was carried out using a 9-point hedonic scale, where 1 corresponded to “dislike very much” and 9 to “like very much”. The evaluated attributes included color, taste, aroma, flavor, and overall acceptability, which were selected because they represent the most relevant sensory quality parameters for consumer acceptance of beverages prepared from coffee or matcha powders packaged in pectin-based films. Sensory evaluation (Figure 6) indicated that the composition of the edible film significantly (*p* < 0.05) affected the perceived quality of the prepared beverages. Among the tested samples, the control beverages prepared from coffee or matcha powder packaged in pectin-based films received the lowest scores for taste, flavor, aroma, and overall acceptability, suggesting that the dissolution of pure pectin in the beverage matrix may have introduced undesirable sensory notes, potentially related to the intrinsic acidity of this hydrocolloid [48]. In comparison, the reference beverages prepared from unpackaged powder exhibited slightly higher acceptability scores than those prepared with control pectin-based films. Color received generally similar scores across all film formulations, irrespective of beverage type. The incorporation of honey into pectin-based films markedly improved sensory performance, with beverages from these formulations achieving high scores for taste, flavor, aroma, and overall acceptability, and being favorably evaluated by the panelists. This improvement is likely attributable to the inherent sweetness, aromatic compounds, and flavor-enhancing properties of honey, which complemented the sensory profile of the beverages. Overall, the matcha beverage received higher sensory scores than the coffee beverage. At concentrations of 0.1%, 0.2%, and 0.3% in edible films, the incorporation of propolis did not significantly influence the perception of sensory attributes, suggesting that the propolis content remained below the level at which consumers perceive the characteristic bitter and resinous notes associated with propolis [49].

## 3. Conclusions

This study successfully developed and characterized edible films based on apple pectin and honey, incorporating propolis as a source of bioactive compounds at concentrations of 0.1%, 0.2%, and 0.3%, and further evaluated their suitability as packaging for soluble coffee and tea powders. The incorporation of honey and propolis modified the physicochemical properties of the films, resulting in increased thickness and moisture content, while reducing mechanical resistance, as reflected in lower tensile strength, elongation at break, and elastic modulus compared to the control pectin film. FT-IR spectra confirmed the presence of the main characteristic peaks across all formulations, while thermal analyses indicated similar degradation patterns, with three distinct stages associated with water loss, decomposition of pectin and honey sugars, and overall matrix degradation. The incorporation of honey and propolis into the films markedly improved the antioxidant composition, as higher concentrations of propolis were directly correlated with increased total phenolic content and antioxidant activity. Furthermore, microbiological analysis confirmed that all films complied with food safety requirements, supporting their potential as safe and functional packaging materials.

When applied as packaging for soluble coffee and matcha powders, the edible films demonstrated additional functional benefits in the prepared beverages. Color analysis showed overall stability across formulations, with only minor variations attributable to the type of film, while propolis incorporation led to increased lightness in coffee beverages. Viscosity measurements indicated that pectin contributed to a thickening effect, whereas the progressive addition of propolis reduced viscosity, likely by interfering with pectin structuring. Notably, both total phenolic content and antioxidant activity of the beverages increased significantly when powders were packaged in pectin–honey–propolis films, confirming the transfer of bioactive compounds from the films into the beverages. Sensory analysis revealed that matcha beverages were generally better accepted than coffee beverages, as films containing honey enhanced flavor, aroma, and overall acceptability. Overall, these findings highlight the potential of pectin-based films enriched with honey and propolis as multifunctional active packaging, combining protective, antioxidant, and sensory-enhancing effects with good consumer acceptance.

## 4. Materials and Methods

### 4.1. Materials

All solvents used were of analytical and HPLC grade. Folin–Ciocalteu reagent, sodium carbonate, and DPPH were obtained from Merck KGaA (Darmstadt, Germany). Apple pectin, methanol, glycerol, aluminum chloride, and the culture medium (plate count agar culture medium, dichloran rose bengal chloramphenicol culture medium, and crystal violet neutral red bile lactose culture medium) used for microbiological analysis were also purchased from Merck KGaA (Darmstadt, Germany).

Polyfloral honey and propolis were purchased from a registered beekeeper in Suceava County, Romania. Two polyfloral honey samples (H1 and H2) from the autumn 2024 harvest were used in this study; despite their common origin, the two samples were expected to differ in physicochemical parameters, allowing evaluation of how honey composition influences the properties of the pectin–honey–propolis films. One raw propolis sample was used in this study. For incorporation into the edible films, the propolis was first frozen at −20 °C for 5 h, then ground using a processor, and finally sieved using a shaker system; the fraction with particle sizes below 50 μm was used for film preparation and kept under freezing conditions to avoid clumping. Freezing and sieving were performed to obtain a fine, homogeneous propolis powder that could be evenly distributed within the pectin–honey film matrix, ensuring reproducible film properties.

### 4.2. Edible Film Preparation

Edible films were prepared using the casting method, following the procedures described previously [7,18,20]. A 3% (*w*/*w*) solution of commercial apple pectin was obtained by dissolving the polysaccharide in ultrapure water under magnetic stirring at 40 °C and 250 rpm for 3 h. The pectin solution was cooled to 25 °C and subsequently mixed with polyfloral honey at a ratio of 80:20 (*w*/*w*); two types of polyfloral honey were used. For pectin–honey–propolis formulations, powdered raw propolis was first incorporated into honey at concentrations of 0.1%, 0.2%, and 0.3% (*w*/*w*, relative to honey) prior to addition to the pectin solution. Glycerol was added at a polymer/glycerol ratio of 0.9:0.1 (*w*/*w*). Control films, containing only apple pectin and glycerol (0.9:0.1, *w*/*w*), were also prepared. All film-forming dispersions were degassed using a vacuum pump, cast onto Teflon plates (150 mm diameter) to achieve a surface solid density of 5.6 mg solids/cm^2^, and dried under controlled conditions (25 °C, 40% relative humidity) for 120 h. The resulting films were conditioned to constant weight in a desiccator containing oversaturated magnesium nitrate solution at 25 °C prior to analysis.

### 4.3. Methods for Physicochemical and Microbiological Analysis of Edible Films

#### 4.3.1. Analysis of Film Thickness and Moisture Content

Film thickness (μm) was measured using a Mitutoyo Absolute thickness gauge (Mitutoyo Corporation, Kanagawa, Japan) in accordance with ASTM Standard Method D6988-21 [50]. The reported values represent the mean of ten measurements taken for each edible film sample.

The moisture content of the film samples was determined according to the ASTM Standard Method D6980-17 [51].

#### 4.3.2. Optical Properties

Optical properties were evaluated in terms of color, film opacity, light transmission, and transparency. The CIE L*a*b* model was used to determine color parameters (lightness, L*; red-green, a*; yellow-blue, b*; chroma, C*_ab_; hue, h*_ab_). The analysis was performed with spectrophotometer CM-5 (Konica Minolta, Tokyo, Japan). The color of films was expressed as the total color difference (ΔE*) according to the following equation [18]:

(1) $$∆E^{*}=\sqrt{(∆L^{*}{)}^{2}+(∆a^{*}{)}^{2}+(∆b^{*}{)}^{2}}$$

where ΔL*, Δa*, and Δb* is the differential between the color parameter of the control sample and the color parameter of the film formulated with pectin, honey, and propolis with different concentrations.

Film opacity was measured according to the methodology reported previously [52], using a spectrophotometer UV-3600 Plus UV-Vis-NIR (Shimadzu Corporation, Kyoto, Japan). The films were cut in rectangular portions and were placed inside the test cell of the spectrophotometer; the blank cell was used as reference. The UV-Vis spectra were recorded at wavelengths from 200 to 600 nm. The opacity was calculated by the following equation [53]:

(2) $$Opacity=\frac{A_{600}}{d}$$

where *A*_600_—absorbance of the film at 600 nm; *d*—film thickness (mm).

The transmittance values (%) were calculated at 600 nm (T_600_). The transparency values were calculated as follows [18]:

(3) $$Transparency=\frac{logT_{600}}{d}$$

where *T*_600_—transmittance (%) at 600 nm; *d*—film thickness (mm).

#### 4.3.3. Mechanical Properties

Mechanical properties were determined using pre-conditioned edible films cut into 100 mm × 25 mm strips, with thickness measured at eight points using a Mitutoyo Absolute thickness gauge (Mitutoyo Corporation, Kanagawa, Japan). Tensile testing was conducted on a Mark-10 ESM301 texture analyzer (Mark-10 Corporation, Copiague, NY, USA) equipped with a 5 kN load cell, by clamping the film ends in the extension grips and stretching at a speed of 10 mm/min until rupture [7,18]. Four replicates were prepared for each film formulation. Force-deformation (N—mm) data were recorded using MESUR^®^gauge Plus software (ver. ESM301, Mark-10 Corporation, Copiague, NY, USA) and converted to stress–Hencky strain curves. Tensile strength (TS), elastic modulus (EM), and percentage of elongation at break (%E) were calculated from these curves.

#### 4.3.4. FT-IR Analysis

Fourier transform infrared (FT-IR) spectroscopy analysis was performed with a Nicolet iS-20 spectrometer (Thermo Scientific, Karlsruhe, Dieselstraße, Germany) to evaluate structural differences among the edible film formulations. Film samples were placed directly onto the ATR crystal, and spectra were collected over the mid-infrared range of 4000–400 cm^−1^ at a spectral resolution of 4 cm^−1^.

#### 4.3.5. Thermal Analysis

Thermal properties of the edible films were evaluated using differential scanning calorimetry (DSC) and thermogravimetric analysis (TGA). For DSC measurements, approximately 2 mg of each sample was hermetically sealed in an aluminum pan with a pinhole and analyzed using a DSC 25 calorimeter (TA Instruments, New Castle, DE, USA). An empty aluminum pan served as the reference. Samples were equilibrated at 25 °C, followed by heating from 0 °C to 300 °C at a constant rate of 10 °C/min under a nitrogen atmosphere, with a purge gas flow rate of 20 mL/min. TGA was performed using TGA 2 Star System (Mettler Toledo, Columbus, OH, USA) by placing approximately 6 mg of the film in an aluminum oxide pan and subjecting it to heating from 25 to 400 °C at a heating rate of 10 °C/min in nitrogen atmosphere (20 mL/min). Sample weight vs. temperature curves were recorded using the Stare software, ver. 17.00 (Mettler Toledo, Columbus, OH, USA).

#### 4.3.6. Determination of Total Phenolic Content, Antioxidant Activity, and Individual Phenolic Compounds

For the determination of total phenolic content (TPC) and antioxidant activity, the films were initially dissolved in distilled water at a concentration of 10 mg/mL and were centrifuged at 4000 rpm for 15 min [22]. The resulting supernatant was used as the test solution. For TPC analysis, 0.2 mL of the test solution was mixed with 2 mL of Folin–Ciocalteu reagent 1:10 and 1.8 mL of sodium carbonate 7.5% (*w*/*v*). The samples were kept in the dark for 30 min and the absorbance was measured at 750 nm using a spectrophotometer UV-3600 Plus UV-Vis-NIR (Shimadzu Corporation, Kyoto, Japan). The results were represented as mg of gallic acid equivalents (GAE) per 100 g of the film.

The antioxidant activity of the films was evaluated using the 2,2-diphenyl-1-picrylhydrazyl (DPPH) radical scavenging assay. For the DPPH assay, the test solution was mixed with the DPPH solution in a 1:3 ration and incubated in the dark for 30 min. Absorbance was then measured at 515 nm using a spectrophotometer UV-3600 Plus UV-Vis-NIR (Shimadzu Corporation, Kyoto, Japan). The DPPH was calculated using the following equation [35]:

(4) $$DPPH\;\left(\%\right)=\left(\frac{A_{0}-A_{1}}{A_{0}}\right)\times 100\;\;$$

where *A*_0_—DPPH absorbance, *A*_1_—sample absorbance.

The individual phenolic compounds in the edible film samples were quantified using high-performance liquid chromatography (HPLC) following a previously described method [35]. Briefly, 1 g of edible film was dissolved in 5 mL of 40% methanol/acidified water (v/v, pH 2, adjusted with HCl) under magnetic stirring at 25 °C and 250 rpm for 10 min. The resulting solution was filtered through 0.45 μm PTFE membrane filters, and a 10 μL aliquot was injected into the HPLC system (Shimadzu, Kyoto, Japan) equipped with a UV SPD-M-20A detector (Shimadzu, Kyoto, Japan). Separation was achieved using a Phenomenex Kinetex Biphenyl 100 Å HPLC column (150 × 4.6 mm, 2.6 μm; Phenomenex Inc., Torrance, CA, USA) thermostated at 25 °C. The mobile phase consisted of 0.1% acetic acid in water (solvent A) and acetonitrile (solvent B), with a flow rate of 1 mL/min. Detection was performed at 280 nm for gallic acid, vanillic acid, protocatechuic acid, and *p*-hydroxybenzoic acid, and at 320 nm for chlorogenic acid, *p*-coumaric acid, caffeic acid, rosmarinic acid, myricetin, quercetin, luteolin, and kaempferol. Calibration curves for all standards exhibited high linearity (R^2^ > 0.99).

#### 4.3.7. Microbiological Analysis

The microbial quality of the edible film samples was evaluated by determining the total viable count, yeast and mold count, and coliform count. For sample preparation, 5 g of each edible film were aseptically weighted and homogenized with 45 mL of 0.1% peptone water (*w*/*v*) to obtain the first dilution (10^−1^). A second dilution (10^−2^) was prepared by transferring 1 mL of the first dilution into 9 mL of 0.1% peptone water. Total viable counts were determined according to ISO 4833-1:2013 [54] using plate count agar (PCA) as the culture medium. Yeast and mold counts were performed using dichloran rose bengal chloramphenicol (DRBC) agar, following ISO 21527-2:2008 [55]. Coliform counts were determined according to ISO 4832:2006 [56] on crystal violet neutral red bile lactose (VRBL) agar as the selective medium, with confirmation in brilliant green lactose bile broth. All materials, equipment, and tools used for microbiological analysis were sterilized prior to use. Sample handling and analytical procedures were performed under aseptic conditions to ensure accurate and reliable microbial enumeration.

### 4.4. Packaging Application of Edible Films

Packaging was carried out using the prepared honey and propolis-based edible films as follows: two pieces measuring 50 × 40 mm were cut from each film and sealed along three of the four edges using the corresponding film-forming solution preheated to 40 °C. The partially sealed packages were conditioned to ensure adequate adhesion of the films, after which they were filled with either 1.5 g of soluble coffee or 1.0 g of tea powder (matcha). The final edge was then sealed using the same procedure. The resulting packaged products measured 50 mm in length, 40 mm in width, and 6 mm in thickness, with a total weight of approximately 4.5 g for the coffee packages and 4.0 g for the tea packages. The packaged samples were stored at 20 °C in a closed container to prevent contamination prior to subsequent analyses.

### 4.5. Physicochemical and Sensory Analysis of Beverages Prepared from Powders Packaged in Edible Films

The solubility of the coffee packaged in edible films was evaluated by immersing the entire package in 150 mL of water with a temperature of 90 °C. For the tea powder packages, solubilization was assessed in 50 mL of water with a temperature of 80 °C.

The coffee and matcha beverages obtained by dissolving soluble coffee and matcha tea powder, respectively, packaged in edible films were analyzed for color, viscosity, total phenolic content, antioxidant activity, and sensory attributes. Color determination was performed as described in Section 4.3.2, except that the samples were placed in a 10 mm-wide cell. Antioxidant activity was assessed by mixing 35 µL of coffee or matcha beverage with 250 µL of DPPH solution, followed by absorbance measurement at 515 nm using a UV-3600 Plus UV-Vis-NIR spectrophotometer (Shimadzu Corporation, Kyoto, Japan). The DPPH scavenging activity was calculated according to the formula described in Section 4.3.6. TPC was determined following the procedure in Section 4.3.6, substituting the coffee or matcha beverage for the test solution.

Viscosity measurements were carried out using a Mars 40 rheometer (Thermo Haake, Burladingen, Germany) equipped with a coaxial cylinder system. Viscosity changes were recorded in controlled rate (CR) mode across a shear rate (γ) range of 0–100 s^−1^.

Sensory evaluation of the coffee and matcha beverages, prepared by dissolving soluble coffee and matcha tea powder packaged in edible films, was performed to assess consumer acceptance based on color, taste, aroma, flavor, and overall acceptability. A 9-point hedonic scale was used, where 1 corresponded to “dislike very much” and 9 to “like very much”. The panel comprised 30 untrained panelists (11 males and 19 females), aged 18–59 years, from the Faculty of Food Engineering, Stefan cel Mare University of Suceava. Evaluations were conducted at 25 °C under uniform white lighting, and samples (20 mL) were served in 40 mL transparent plastic cups coded with random three-digit numbers.

### 4.6. Statistical Analysis

The data obtained from the analysis of the physicochemical properties were subjected to analysis of variance (ANOVA) using OriginPro 2025b software, trial version (OriginLab Corporation, Northampton, MA, USA) and IBM SPSS Statistics v.26, trial version (IBM, Chicago, IL, USA). Differences between means were evaluated using Fisher’s Least Significant Difference (LSD) test at a 95% confidence level.

## Figures and Tables

**Table 1:** Physical, optical and mechanical properties of edible films based on pectin (P), honey (H1 and H2) and propolis (Pr). Mean values, with standard deviations in parentheses.

Parameter	Edible Film Formulation								
	P	P–H1	P–H1–Pr 0.1	P–H1–Pr 0.2	P–H1–Pr 0.3	P–H2	P–H2–Pr 0.1	P–H2–Pr 0.2	P–H2–Pr 0.3
Thickness (μm)	70.50 (2.30) ^d^	255.80 (1.10) ^c^	261.10 (3.30) ^bc^	267.90 (2.90) ^ab^	275.60 (2.70) ^a^	259.20 (2.00) ^bc^	269.50 (1.90) ^ab^	274.30 (4.10) ^a^	276.90 (7.70) ^a^
Moisture content (%)	10.05 (1.24) ^b^	17.23 (0.01) ^a^	16.99 (0.01) ^a^	16.95 (0.05) ^a^	17.07 (0.02) ^a^	17.25 (0.03) ^a^	17.57 (0.59) ^a^	16.82 (0.13) ^a^	17.25 (0.06) ^a^
Optical properties									
L*	38.00 (0.09) ^a^	32.32 (0.52) ^c^	31.58 (0.04) ^d^	31.21 (0.01) ^d^	27.49 (0.06) ^g^	33.08 (0.09) ^b^	31.58 (0.07) ^d^	30.57 (0.17) ^e^	29.89 (0.27) ^f^
a*	−0.05 (0.03) ^a^	−0.12 (0.03) ^a^	−0.47 (0.02) ^c^	−0.74 (0.04) ^e^	−0.61 (0.01) ^d^	−0.47 (0.02) ^c^	−0.53 (0.03) ^c^	−0.46 (0.01) ^c^	−0.31 (0.01) ^b^
b*	2.63 (0.03) ^c^	2.81 (0.03) ^b^	2.36 (0.05) ^e^	3.25 (0.02) ^a^	2.45 (0.04) ^e^	2.60 (0.02) ^cd^	3.18 (0.02) ^a^	3.22 (0.07) ^a^	2.48 (0.05) ^de^
ΔE*	–	4.93 (0.03) ^f^	5.70 (0.58) ^e^	6.49 (0.07) ^d^	6.81 (0.09) ^d^	6.43 (0.05) ^d^	7.47 (0.08) ^c^	8.14 (0.18) ^b^	10.52 (0.03) ^a^
C*_ab_	2.63 (0.03) ^d^	2.81 (0.03) ^c^	2.41 (0.05) ^f^	3.33 (0.02) ^a^	2.52 (0.05) ^e^	2.64 (0.02) ^d^	3.23 (0.02) ^b^	3.23 (0.04) ^b^	2.50 (0.05) ^ef^
h*_ab_	91.02 (0.46) ^g^	92.47 (0.62) ^g^	101.43 (0.69) ^bc^	102.85 (0.53) ^ab^	104.07 (0.76) ^a^	100.18 (0.23) ^cd^	99.42 (0.44) ^de^	98.23 (0.38) ^ef^	97.20 (0.30) ^f^
Opacity	8.76 (0.29) ^a^	1.87 (0.01) ^d^	1.87 (0.02) ^d^	2.27 (0.02) ^c^	2.51 (0.03) ^c^	1.35 (0.01) ^e^	2.92 (0.02) ^b^	2.86 (0.04) ^b^	3.05 (0.08) ^b^
Light transmission (%)	24.14 (0.01) ^c^	33.17 (0.01) ^b^	32.52 (0.01) ^b^	21.30 (0.01) ^d^	14.68 (0.72) ^f^	44.70 (0.01) ^a^	16.51 (0.01) ^e^	16.45 (0.01) ^e^	14.30 (0.01) ^f^
Transparency	19.62 (0.65) ^a^	5.94 (0.03) ^b^	5.79 (0.07) ^b^	4.96 (0.05) ^c^	4.23 (0.05) ^d^	6.36 (0.05) ^b^	4.52 (0.03) ^cd^	4.43 (0.07) ^cd^	4.17 (0.11) ^d^
Mechanical properties									
EM (MPa)	9.79 (0.12) ^a^	2.96 (0.13) ^b^	2.83 (0.01) ^b^	2.78 (0.16) ^b^	2.14 (0.27) ^c^	2.69 (0.02) ^b^	2.64 (0.02) ^b^	2.48 (0.09) ^bc^	2.19 (0.02) ^c^
TS (MPa)	5.81 (0.07) ^a^	1.18 (0.03) ^b^	1.08 (0.03) ^b^	0.69 (0.04) ^cd^	0.59 (0.02) ^c^	1.22 (0.04) ^b^	0.97 (0.01) ^b^	0.78 (0.01) ^c^	0.73 (0.01) ^c^
%E	23.60 (0.25) ^c^	34.49 (0.12) ^a^	33.68 (0.24) ^a^	28.76 (0.12) ^b^	23.72 (0.4) ^c^	33.37 (0.66) ^a^	32.48 (0.2) ^ab^	30.32 (0.16) ^b^	29.09 (0.36) ^b^

^a–g^ Different letters in the same line indicate significant differences among samples (*p* < 0.05); P—pectin, H—honey, Pr—propolis; EM—elastic modulus, TS—tensile strength, %E—elongation at break.

**Table 2:** DSC parameters of edible films with pectin, honey and propolis. Mean values, with standard deviations in parentheses.

Edible Film Formulation	Tm_1_ (°C)	ΔH (J/g)	Tm_2_ (°C)	ΔH (J/g)	Td (°C)	ΔCp (J/(g. °C))	ΔH (J/g)
P	146.41 (1.24) ^d^	6.06 (0.05) ^a^	198.46 (3.32) ^a^	117.96 (2.64) ^f^	227.59 (1.98) ^c^	0.61 (0.00) ^b^	20.19 (0.60) ^b^
P–H1	149.17 (1.98) ^c^	4.43 (0.02) ^c^	177.78 (2.55) ^c^	305.68 (2.38) ^b^	259.57 (2.60) ^a^	0.14 (0.00) ^d^	31.85 (1.14) ^a^
P–H1–Pr 0.1	150.64 (1.29) ^b^	6.69 (0.10) ^a^	177.50 (1.73) ^c^	270.05 (2.29) ^cd^	230.87 (4.88) ^b^	0.23 (0.00) ^d^	12.13 (1.73) ^c^
P–H1–Pr 0.2	148.46 (2.44) ^c^	6.39 (0.14) ^a^	182.60 (2.66) ^b^	287.68 (3.66) ^c^	235.97 (2.62) ^b^	0.47 (0.01) ^b^	11.56 (1.08) ^c^
P–H1–Pr 0.3	150.16 (1.89) ^b^	6.04 (0.20) ^a^	186.11 (1.85) ^b^	325.35 (2.89) ^a^	225.66 (3.11) ^c^	1.07 (0.03) ^a^	5.86 (0.23) ^d^
P–H2	152.72 (1.49) ^a^	3.64 (0.06) ^d^	159.41 (1.83) ^e^	263.85 (3.24) ^d^	238.89 (2.63) ^b^	0.32 (0.00) ^c^	28.02 (1.64) ^a^
P–H2–Pr 0.1	137.89 (1.80) ^d^	5.18 (0.16) ^b^	159.22 (2.68) ^e^	170.50 (2.97) ^e^	238.10 (2.85) ^b^	0.35 (0.00) ^c^	15.26 (1.34) ^c^
P–H2–Pr 0.2	151.77 (1.76) ^a^	5.03 (0.08) ^b^	169.42 (3.04) ^d^	261.64 (2.29) ^d^	223.27 (2.62) ^c^	0.55 (0.01) ^b^	13.19 (0.62) ^c^
P–H2–Pr 0.3	150.57 (0.72) ^b^	4.34 (0.10) ^c^	183.34 (1.63) ^b^	296.84 (4.25) ^b^	236.32 (3.00) ^b^	1.11 (0.02) ^a^	12.91 (1.29) ^c^

^a–f^ Different letters in the same column indicate significant differences among samples (*p* < 0.05); P—pectin, H—honey, Pr—propolis.

**Table 3:** TGA parameters of edible films with pectin, honey and propolis. Mean values, with standard deviations in parentheses.

Edible Film Formulation	I Stage of Weight Loss			II Stage of Weight Loss			III Stage of Weight Loss		
	T_peak_ (°C)	ΔW (%)	Residue at 150 °C (%)	T_peak_ (°C)	ΔW (%)	Residue at 280 °C (%)	T_peak_ (°C)	ΔW (%)	Residue at 400 °C (%)
P	105.08 (0.84) ^a^	12.39 (0.08) ^a^	87.62 (0.08) ^g^	210.72 (0.88) ^d^	33.03 (0.01) ^a^	54.59 (0.09) ^e^	362.99 (0.81) ^a^	2.64 (0.06) ^d^	51.95 (0.16) ^ef^
P–H1	92.93 (0.13) ^ef^	10.85 (0.90) ^b^	89.16 (0.90) ^f^	200.05 (0.37) ^f^	25.92 (0.06) ^b^	63.24 (0.96) ^d^	342.65 (0.84) ^e^	13.42 (0.06) ^a^	49.82 (1.02) ^fg^
P–H1–Pr 0.1	96.38 (0.35) ^cd^	6.81 (0.10) ^ef^	93.19 (0.10) ^bc^	189.77 (0.38) ^h^	24.09 (0.03) ^c^	69.10 (0.13) ^b^	347.95 (1.43) ^c^	11.35 (0.16) ^b^	57.76 (0.29) ^bc^
P–H1–Pr 0.2	94.94 (0.11) ^d^	7.80 (0.08) ^de^	92.21 (0.08) ^cd^	228.21 (0.34) ^b^	24.52 (0.53) ^c^	67.69 (0.61) ^bc^	341.96 (0.27) ^e^	10.83 (0.21) ^b^	56.86 (0.82) ^cd^
P–H1–Pr 0.3	98.62 (0.20) ^b^	8.62 (0.23) ^cd^	91.38 (0.23) ^de^	184.10 (0.83) ^i^	23.80 (0.11) ^cd^	67.58 (0.11) ^bc^	346.95 (0.30) ^cd^	8.54 (0.59) ^c^	59.04 (0.71) ^abc^
P–H2	97.82 (0.23) ^bc^	12.79 (0.13) ^a^	87.21 (0.13) ^g^	194.01 (0.10) ^g^	25.81 (0.47) ^b^	61.41 (0.60) ^d^	329.97 (0.21) ^f^	13.62 (0.09) ^a^	47.79 (0.69) ^g^
P–H2–Pr 0.1	94.80 (0.32) ^de^	4.70 (0.27) ^g^	95.30 (0.27) ^a^	222.68 (0.67) ^c^	23.09 (0.04) ^de^	72.21 (0.31) ^a^	343.60 (0.79) ^de^	11.56 (0.59) ^b^	60.65 (0.91) ^a^
P–H2–Pr 0.2	90.52 (0.48) ^g^	5.71 (0.19) ^fg^	94.30 (0.19) ^ab^	231.33 (0.76) ^a^	22.31 (0.08) ^e^	71.99 (0.27) ^a^	355.29 (1.20) ^b^	11.82 (0.03) ^b^	60.17 (0.30) ^ab^
P–H2–Pr 0.3	91.14 (1.02) ^fg^	9.95 (0.25) ^bc^	90.05 (0.25) ^ef^	205.80 (1.03) ^e^	23.96 (0.20) ^cd^	66.09 (0.45) ^c^	327.93 (1.66) ^f^	11.66 (0.05) ^b^	54.44 (0.50) ^de^

^a–i^ Different letters in the same column indicate significant differences among samples (*p* < 0.05); P—pectin, H—honey, Pr—propolis. The residue values at 150 °C, 280 °C, and 400 °C represent the amount of material that remains undecomposed at each stage of thermal degradation.

**Table 4:** Total phenolic content, phenolic profile, and antioxidant activity of edible films with pectin, honey and propolis. Mean values, with standard deviations in parentheses.

Parameter	Edible Film Formulation								
	P	P–H1	P–H1–Pr 0.1	P–H1–Pr 0.2	P–H1–Pr 0.3	P–H2	P–H2–Pr 0.1	P–H2–Pr 0.2	P–H2–Pr 0.3
TPC (mg GAE/100 g)	3.02 (0.05) ^f^	42.61 (0.62) ^d^	46.60 (0.67) ^cd^	50.94 (0.73) ^b^	54.21 (0.80) ^a^	41.09 (0.59) ^d^	45.19 (0.65) ^d^	48.31 (0.69) ^c^	52.90 (0.76) ^ab^
DPPH (%)	14.71 (0.21) ^e^	55.29 (0.05) ^c^	57.27 (0.05) ^b^	59.07 (0.10) ^a^	59.91 (0.05) ^a^	53.77 (0.08) ^d^	54.56 (0.05) ^cd^	56.75 (0.02) ^c^	59.89 (0.78) ^a^
Individual polyphenols content (mg/100 g)									
Galic acid	n.d.	n.d.	n.d.	n.d.	n.d.	n.d.	n.d.	n.d.	n.d.
Protocatechuic acid	n.d.	n.d.	0.34 (0.48) ^a^	0.44 (0.27) ^a^	0.84 (0.62) ^a^	n.d.	0.22 ^b^	0.32 (0.16) ^a^	0.99 (0.45) ^a^
4-hydroxybenzoic acid	n.d.	37.90 (2.54) ^c^	36.74 (9.98) ^c^	35.63 (9.94) ^c^	34.85 (10.85) ^c^	45.31 (1.36) ^a^	44.29 (2.11) ^a^	43.61 (11.27) ^b^	42.84 (11.12) ^b^
Vanillic acid	n.d.	n.d.	0.86 (0.01) ^a^	0.98 (0.05) ^a^	1.44 (2.03) ^a^	n.d.	n.d.	n.d.	n.d.
Caffeic acid	n.d.	23.48 (0.01) ^c^	23.60 (0.04) ^c^	24.28 (0.13) ^c^	26.86 (0.10) ^b^	26.33 (0.15) ^b^	26.77 (0.25) ^b^	26.86 (0.51) ^b^	28.99 (0.14) ^a^
Chlorogenic acid	n.d.	n.d.	n.d.	n.d.	n.d.	n.d.	n.d.	n.d.	n.d.
*p*-coumaric acid	n.d.	0.05 (0.00) ^e^	25.60 (0.41) ^d^	29.89 (0.59) ^c^	34.62 (0.08) ^b^	0.06 (0.00) ^e^	32.78 (2.84) ^bc^	34.98 (0.12) ^b^	40.81 (0.12) ^a^
Rosmarinic acid	n.d.	0.25 (0.01) ^d^	0.29 (0.01) ^c^	0.31 (0.01) ^c^	0.32 (0.01) ^c^	0.33 (0.00) ^c^	0.36 (0.01) ^b^	0.38 (0.00) ^b^	0.71 (0.01) ^a^
Miricetin	n.d.	6.22 (0.06) ^d^	10.08 (0.08) ^ab^	10.86 (0.06) ^a^	11.15 (0.07) ^a^	7.37 (3.12) ^cd^	8.35 (0.05) ^c^	9.41 (0.05) ^b^	9.57 (0.09) ^b^
Luteolin	n.d.	n.d.	n.d.	n.d.	n.d.	n.d.	n.d.	n.d.	n.d.
Quercitin	n.d.	1.09 ^e^	1.11 (0.03) ^d^	1.18 (0.08) ^d^	1.26 (0.04) ^c^	1.28 (0.04) ^c^	1.39 (0.01) ^bc^	1.44 (0.03) ^b^	1.61 (0.03) ^a^
Kaempferol	n.d.	n.d.	0.77 (0.17) ^d^	1.31 (0.01) ^c^	1.90 (0.07) ^b^	n.d.	1.78 (0.20) ^b^	1.87 (0.45) ^b^	2.30 (0.01) ^a^

^a–e^ Different letters in the same line indicate significant differences among samples (*p* < 0.05); n.d.—not detected; P—pectin, H—honey, Pr—propolis.

**Table 5:** Microbiological quality parameters of edible films. Mean values, with standard deviations in parentheses.

Edible Film Formulation	TVC (log_10_ CFU/g)	YMC (log_10_ CFU/g)	CC (log_10_ CFU/g)
P	1.76 (0.03) ^a^	1.31 (0.01) ^b^	n.d.
P–H1	1.79 (0.02) ^a^	1.40 (0.01) ^a^	n.d.
P–H1–Pr 0.1	1.78 (0.01) ^a^	1.38 (0.03) ^a^	n.d.
P–H1–Pr 0.2	1.78 (0.01) ^a^	1.36 (0.03) ^ab^	n.d.
P–H1–Pr 0.3	1.77 (0.01) ^a^	1.36 (0.01) ^ab^	n.d.
P–H2	1.78 (0.03) ^a^	1.41 (0.03) ^a^	n.d.
P–H2–Pr 0.1	1.78 (0.01) ^a^	1.39 (0.01) ^a^	n.d.
P–H2–Pr 0.2	1.77 (0.01) ^a^	1.38 (0.01) ^a^	n.d.
P–H2–Pr 0.3	1.77 (0.02) ^a^	1.37 (0.02) ^a^	n.d.

^a,b^ Different letters in the same column indicate significant differences among samples (*p* < 0.05). TVC—total viable count, YMC—yeast and mold count, CC—coliform count, n.d.—not detected, P—pectin, H—honey, Pr—propolis.

**Table 6:** Physicochemical properties of beverages formulated from powders packaged in edible films. Mean values, with standard deviations in parentheses.

Film Used as Package	Color Parameters						Viscosity (Pa∙s)	TPC (mg GAE/100 g)	DPPH (%)
	L*	a*	b*	ΔE*	C*_ab_	h*_ab_			
Coffee beverage formulated from powder packaged in edible film									
no film	48.66 (0.03) ^g^	29.99 (0.01) ^b^	74.69 (0.07) ^d^	–	80.49 (0.06) ^c^	68.12 (0.03) ^h^	0.71 × 10^−3^ (0.00) ^e^	233.86 (3.34) ^d^	50.15 (0.07) ^d^
P	50.24 (0.07) ^e^	28.85 (0.21) ^c^	76.08 (0.04) ^b^	2.40 (0.24) ^de^	81.42 (0.04) ^b^	69.15 (0.02) ^e^	1.61 × 10^−3^ (0.00) ^a^	238.23 (3.40) ^d^	53.77 (0.07) ^d^
P–H1	51.08 (0.01) ^c^	28.45 (0.01) ^d^	76.62 (0.12) ^a^	3.45 (0.01) ^abc^	81.73 (0.11) ^a^	69.63 (0.02) ^cd^	1.60 × 10^−3^ (0.01) ^a^	249.93 (3.57) ^c^	58.18 (0.07) ^b^
P–H1–Pr 0.1	48.39 (0.02) ^h^	28.64 (0.01) ^cd^	73.04 (0.07) ^f^	2.15 (0.10) ^e^	78.45 (0.07) ^ef^	68.59 (0.01) ^g^	1.46 × 10^−3^ (0.04) ^b^	273.94 (3.92) ^b^	60.64 (0.00) ^b^
P–H1–Pr 0.2	48.18 (0.01) ^i^	30.27 (0.01) ^a^	74.75 (0.03) ^d^	0.56 (0.01) ^f^	80.64 (0.03) ^c^	67.96 (0.01) ^i^	1.24 × 10^−3^ (0.01) ^d^	284.60 (4.07) ^a^	63.71 (0.07) ^a^
P–H1–Pr 0.3	50.77 (0.01) ^d^	27.3 (0.00) ^f^	73.77 (0.02) ^e^	3.54 (0.01) ^abc^	78.66 (0.02) ^e^	69.69 (0.00) ^c^	1.21 × 10^−3^ (0.02) ^d^	293.21 (4.19) ^a^	66.62 (0.07) ^a^
P–H2	51.36 (0.01) ^b^	27.31 (0.01) ^f^	74.84 (0.10) ^d^	3.81 (0.02) ^ab^	79.67 (0.09) ^d^	69.96 (0.02) ^b^	1.59 × 10^−3^ (0.01) ^a^	238.78 (3.41) ^d^	56.48 (0.07) ^c^
P–H2–Pr 0.1	49.22 (0.01) ^f^	28.03 (0.01) ^e^	73.00 (0.09) ^f^	2.66 (0.09) ^cde^	78.19 (0.08) ^f^	69.00 (0.02) ^f^	1.47 × 10^−3^ (0.01) ^b^	253.60 (3.62) ^c^	59.29 (0.07) ^b^
P–H2–Pr 0.2	52.54 (0.01) ^a^	27.43 (0.01) ^f^	75.67 (0.11) ^c^	4.27 (0.66) ^a^	80.49 (0.10) ^c^	70.08 (0.03) ^a^	1.39 × 10^−3^ (0.01) ^c^	265.18 (3.79) ^bc^	60.79 (0.07) ^b^
P–H2–Pr 0.3	51.27 (0.01) ^b^	28.17 (0.01) ^e^	75.65 (0.01) ^c^	3.32 (0.03) ^bcd^	80.72 (0.00) ^c^	69.58 (0.01) ^d^	1.14 × 10^−3^ (0.02) ^de^	286.96 (4.09) ^a^	64.61 (0.07) ^a^
Matcha beverage formulated from powder packaged in edible film									
no film	54.67 (1.09) ^bc^	−3.85 (0.11) ^d^	43.86 (0.12) ^bc^	–	44.05 (0.13) ^bc^	95.00 (0.12) ^b^	1.06 × 10^−3^ (0.01) ^d^	282.01 (4.03) ^e^	57.08 (0.07) ^d^
P	46.12 (0.76) ^e^	−2.61 (0.12) ^a^	44.26 (0.25) ^ab^	8.65 (0.34) ^a^	44.33 (0.25) ^ab^	93.38 (0.14) ^de^	2.17 × 10^−3^ (0.04) ^a^	285.86 (4.08) ^e^	58.59 (0.07) ^d^
P–H1	61.01 (0.67) ^a^	−4.84 (0.05) ^e^	41.33 (0.18) ^e^	6.90 (0.29) ^bc^	41.61 (0.17) ^e^	96.67 (0.09) ^a^	2.13 × 10^−3^ (0.01) ^a^	298.48 (4.26) ^d^	63.51 (0.07) ^c^
P–H1–Pr 0.1	47.69 (1.44) ^de^	−2.56 (0.17) ^a^	43.94 (0.37) ^bc^	7.10 (0.35) ^ab^	44.00 (0.39) ^bc^	93.33 (0.18) ^e^	2.02 × 10^−3^ (0.02) ^b^	315.24 (4.50) ^c^	67.02 (0.07) ^b^
P–H1–Pr 0.2	51.15 (0.92) ^cd^	−2.77 (0.08) ^ab^	44.79 (0.23) ^a^	3.79 (0.19) ^de^	44.87 (0.23) ^a^	93.54 (0.09) ^de^	1.87 × 10^−3^ (0.00) ^bc^	334.24 (4.78) ^b^	68.02 (0.07) ^b^
P–H1–Pr 0.3	55.1 (0.70) ^b^	−2.83 (0.06) ^abc^	43.01 (0.10) ^d^	1.43 (0.07) ^fg^	43.10 (0.10) ^d^	93.77 (0.06) ^cd^	1.61 × 10^−3^ (0.02) ^c^	373.82 (5.35) ^a^	70.13 (0.07) ^a^
P–H2	59.89 (0.01) ^a^	−3.80 (0.00) ^d^	43.21 (0.01) ^cd^	5.24 (1.06) ^cd^	43.38 (0.01) ^cd^	95.03 (0.01) ^b^	2.16 × 10^−3^ (0.02) ^a^	287.17 (4.10) ^e^	62.05 (0.14) ^c^
P–H2–Pr 0.1	54.22 (1.24) ^bc^	−3.18 (0.05) ^c^	43.89 (0.08) ^bc^	0.82 (0.04) ^g^	44.00 (0.09) ^bc^	94.14 (0.05) ^c^	2.05 × 10^−3^ (0.01) ^b^	311.19 (4.44) ^c^	65.21 (0.07) ^c^
P–H2–Pr 0.2	52.38 (0.98) ^bc^	−3.01 (0.08) ^bc^	43.44 (0.19) ^cd^	2.48 (0.10) ^efg^	43.54 (0.20) ^bcd^	93.97 (0.09) ^c^	1.91 × 10^−3^ (0.00) ^b^	327.28 (4.67) ^bc^	66.92 (0.07) ^b^
P–H2–Pr 0.3	51.91 (0.74) ^bc^	−3.13 (0.08) ^bc^	43.75 (0.16) ^bcd^	2.86 (0.35) ^ef^	43.85 (0.16) ^bcd^	94.09 (0.08) ^c^	1.75 × 10^−3^ (0.00) ^c^	342.65 (4.89) ^b^	69.63 (0.07) ^a^

^a–i^ Different letters in the same column indicate significant differences among samples (*p* < 0.05); P—pectin, H—honey, Pr—propolis.

## Data Availability

The original contributions presented in this study are included in the article. Further inquiries can be directed to the corresponding authors.

## References

[1] Syarifuddin A., Muflih M.H., Izzah N., Fadillah U., Ainani A.F., Dirpan A. (2025). Pectin-Based Edible Films and Coatings: From Extraction to Application on Food Packaging towards Circular Economy- A Review. Carbohydr. Polym. Technol. Appl..

[2] Zhang X., Chen X., Dai J., Cui H., Lin L. (2024). Edible Films of Pectin Extracted from Dragon Fruit Peel: Effects of Boiling Water Treatment on Pectin and Film Properties. Food Hydrocoll..

[3] Gaspar M.C., Braga M.E.M. (2023). Edible Films and Coatings Based on Agrifood Residues: A New Trend in the Food Packaging Research. Curr. Opin. Food Sci..

[4] Kocira A., Kozłowicz K., Panasiewicz K., Staniak M., Szpunar-Krok E., Hortyńska P. (2021). Polysaccharides as Edible Films and Coatings: Characteristics and Influence on Fruit and Vegetable Quality—A Review. Agronomy.

[5] Biratu G., Woldemariam H.W., Gonfa G. (2024). Development of Active Edible Films from Coffee Pulp Pectin, Propolis, and Honey with Improved Mechanical, Functional, Antioxidant, and Antimicrobial Properties. Carbohydr. Polym. Technol. Appl..

[6] Umaraw P., Verma A.K. (2017). Comprehensive Review on Application of Edible Film on Meat and Meat Products: An Eco-Friendly Approach. Crit. Rev. Food Sci. Nutr..

[7] Dranca F., Talón E., Vargas M., Oroian M. (2021). Microwave vs. Conventional Extraction of Pectin from Malus Domestica ‘Fălticeni’ Pomace and Its Potential Use in Hydrocolloid-Based Films. Food Hydrocoll..

[8] Mulla M.Z., Ahmed J., Vahora A., Pathania S. (2023). Effect of Pectin Incorporation on Characteristics of Chitosan Based Edible Films. J. Food Meas. Charact..

[9] Priyadarshi R., Kim S.M., Rhim J.W. (2021). Pectin/Pullulan Blend Films for Food Packaging: Effect of Blending Ratio. Food Chem..

[10] Kannan A., Dheeptha M., Sistla Y.S. (2023). Development of Pectin and Sodium Alginate Composite Films with Improved Barrier and Mechanical Properties for Food-Packaging Applications. Eng. Proc..

[11] Dao D.N., Le P.H., Do D.X., Dang T.M.Q., Nguyen S.K., Nguyen V. (2023). Pectin and Cellulose Extracted from Coffee Pulps and Their Potential in Formulating Biopolymer Films. Biomass Convers. Biorefinery.

[12] Asfaw W.A., Tafa K.D., Satheesh N. (2023). Optimization of Citron Peel Pectin and Glycerol Concentration in the Production of Edible Film Using Response Surface Methodology. Heliyon.

[13] Šešlija S., Nešić A., Ružić J., Kalagasidis Krušić M., Veličković S., Avolio R., Santagata G., Malinconico M. (2018). Edible Blend Films of Pectin and Poly(Ethylene Glycol): Preparation and Physico-Chemical Evaluation. Food Hydrocoll..

[14] Chuenkaek T., Kobayashi T. (2024). Citrus Waste Upcycling toward Pectin Moisturizer Films Plasticized with Glycerol and Polyethylene Glycol. ACS Sustain. Resour. Manag..

[15] Auras R., Arroyo B., Selke S. (2009). Production and Properties of Spin-Coated Cassava-Starch-Glycerol-Beeswax Films. Starch Stärke.

[16] Vergel-Alfonso A.A., Arias-Avelenda R., Casariego-Año A., Giménez M.J., Ruíz-Cruz S., López-Corona B.E., Del-Toro-Sánchez C.L., Gonzalez-Bravo A.L., Plascencia-Jatomea M., Menchaca-Armenta M. (2025). Development and Characterization of Pectin and Beeswax-Based Coatings Enhanced with Anthocyanins and Its Antioxidant and Antifungal Properties. Process.

[17] Osuna M.B., Romero C.A., Rivas F.P., Judis M.A., Bertola N.C. (2025). Apple Pectin Based Film with Apis Mellifera Honey and /or Propolis Extract as Sources of Active Compounds. Food Biophys..

[18] Spinei M., Oroian M., Ursachi V.F. (2024). Characterization of Biodegradable Films Based on Carboxymethyl Cellulose and Citrus Pectin Films Enriched with Bee Bread Oil and Thyme Oil. LWT.

[19] Younis H.G.R., Zhao G. (2019). Physicochemical Properties of the Edible Films from the Blends of High Methoxyl Apple Pectin and Chitosan. Int. J. Biol. Macromol..

[20] Ursachi V.F., Oroian M., Spinei M. (2024). Development and Characterization of Biodegradable Films Based on Cellulose Derivatives and Citrus Pectin: A Comparative Study. Ind. Crops Prod..

[21] Ran R., Xiong Y., Zheng T., Tang P., Zhang Y., Yang C., Li G. (2024). Active and Intelligent Collagen Films Containing Laccase-Catalyzed Mulberry Extract and Pickering Emulsion for Fish Preservation and Freshness Indicator. Food Hydrocoll..

[22] Li X., He J., Zhang W., Khan M.R., Ahmad N., Tian W. (2024). Pectin Film Fortified with Zein Nanoparticles and Fe3+-Encapsulated Propolis Extract for Enhanced Fruit Preservation. Food Hydrocoll..

[23] Marangoni Júnior L., Gonçalves S.d.Á., Silva R.G.d., Martins J.T., Vicente A.A., Alves R.M.V., Vieira R.P. (2022). Effect of Green Propolis Extract on Functional Properties of Active Pectin-Based Films. Food Hydrocoll..

[24] Jridi M., Abdelhedi O., Salem A., Kechaou H., Nasri M., Menchari Y. (2020). Physicochemical, Antioxidant and Antibacterial Properties of Fish Gelatin-Based Edible Films Enriched with Orange Peel Pectin: Wrapping Application. Food Hydrocoll..

[25] Osuna M.B., Michaluk A., Romero A.M., Judis M.A., Bertola N.C. (2022). Plasticizing Effect of Apis Mellifera Honey on Whey Protein Isolate Films. Biopolymers.

[26] Gniewosz M., Pobiega K., Kraśniewska K., Synowiec A., Chaberek M., Galus S. (2022). Characterization and Antifungal Activity of Pullulan Edible Films Enriched with Propolis Extract for Active Packaging. Foods.

[27] Bodini R.B., Sobral P.J.A., Favaro-Trindade C.S., Carvalho R.A. (2013). Properties of Gelatin-Based Films with Added Ethanol–Propolis Extract. LWT—Food Sci. Technol..

[28] Pauliuc D., Dranca F., Ropciuc S., Oroian M. (2022). Advanced Characterization of Monofloral Honeys from Romania. Agriculture.

[29] Dranca F., Vargas M., Oroian M. (2020). Physicochemical Properties of Pectin from Malus Domestica ‘Fălticeni’ Apple Pomace as Affected by Non-Conventional Extraction Techniques. Food Hydrocoll..

[30] Oroian M., Ursachi F., Dranca F. (2020). Influence of Ultrasonic Amplitude, Temperature, Time and Solvent Concentration on Bioactive Compounds Extraction from Propolis. Ultrason. Sonochem..

[31] Velásquez P., Montenegro G., Valenzuela L.M., Giordano A., Cabrera-Barjas G., Martin-Belloso O. (2022). K-Carrageenan Edible Films for Beef: Honey and Bee Pollen Phenolic Compounds Improve Their Antioxidant Capacity. Food Hydrocoll..

[32] Moreira R.B., Teixeira J.A., Furuyama-Lima A.M., De Souza N.C., Siqueira A.B. (2014). Preparation, Characterization and Evaluation of Drug-Delivery Systems: Pectin and Mefenamic Acid Films. Thermochim. Acta.

[33] Marcucci M.C., Cunha I.B.S., Sanchez E.M.S., Gonçalves C.P., Cedeño-Pinos C., Bañón S. (2022). Analysis of Brazilian Propolis by Differential Scanning Calorimetry (DSC) and Thermal Gravimetric Analysis (TGA). Characteristics of Crude Resin, Ethanolic Extracts, Wax and Isolated Compounds. Bee World.

[34] Kamthai S., Wiriyacharee P., Naruenartwongsakul S., Khaw-on P., Deenu A., Chaipoot S., Phongphisutthinant R., Tachai K., Orpool S. (2025). Influence of Honey Bee Brood Protein on the Hydrophilic, Mechanical, and Thermal Properties of Polysaccharide Gel Films. Gels.

[35] Pauliuc D., Dranca F., Oroian M. (2020). Antioxidant Activity, Total Phenolic Content, Individual Phenolics and Physicochemical Parameters Suitability for Romanian Honey Authentication. Foods.

[36] Tlak Gajger I., Dar S.A., Ahmed M.M.M., Aly M.M., Vlainić J. (2025). Antioxidant Capacity and Therapeutic Applications of Honey: Health Benefits, Antimicrobial Activity and Food Processing Roles. Antioxidants.

[37] Woźniak M., Sip A., Mrówczyńska L., Broniarczyk J., Waśkiewicz A., Ratajczak I. (2023). Biological Activity and Chemical Composition of Propolis from Various Regions of Poland. Molecules.

[38] Díaz-Montes E., Castro-Muñoz R. (2021). Edible Films and Coatings as Food-Quality Preservers: An Overview. Foods.

[39] Gomes S., Dias L.G., Moreira L.L., Rodrigues P., Estevinho L. (2010). Physicochemical, Microbiological and Antimicrobial Properties of Commercial Honeys from Portugal. Food Chem. Toxicol..

[40] Rendueles E., Mauriz E., Sanz-Gómez J., Adanero-Jorge F., García-Fernandez C. (2023). Antimicrobial Activity of Spanish Propolis against Listeria Monocytogenes and Other Listeria Strains. Microorganisms.

[41] Vică M.L., Glevitzky M., Tit D.M., Behl T., Heghedűş-Mîndru R.C., Zaha D.C., Ursu F., Popa M., Glevitzky I., Bungău S. (2021). The Antimicrobial Activity of Honey and Propolis Extracts from the Central Region of Romania. Food Biosci..

[42] European Commission COMMISSION REGULATION (EC) No 2073/2005. https://eur-lex.europa.eu/legal-content/EN/TXT/HTML/?uri=CELEX:02005R2073-20200308.

[43] Najman K., Sadowska A., Wolińska M., Starczewska K., Buczak K. (2023). The Content of Bioactive Compounds and Technological Properties of Matcha Green Tea and Its Application in the Design of Functional Beverages. Molecules.

[44] Manikharda, Shofi V.E., Betari B.K., Supriyadi (2023). Effect Shading Intensity on Color, Chemical Composition, and Sensory Evaluation of Green Tea (*Camelia Sinensis Var Assamica*). J. Saudi Soc. Agric. Sci..

[45] Behfar M., Hashemirad F.S., Kavoosi G., Dadfar S.M.M. (2025). Enhancing Gelatin Matrices with Propolis and Royal Jelly: Antioxidant, Physico-Chemical, Techno-Functional, and Physico-Mechanical Properties. J. Agric. Food Res..

[46] Jakubczyk K., Kochman J., Kwiatkowska A., Kałdunska J., Dec K., Kawczuga D., Janda K. (2020). Antioxidant Properties and Nutritional Composition of Matcha Green Tea. Foods.

[47] Pintać D., Bekvalac K., Mimica-Dukić N., Rašeta M., Anđelić N., Lesjak M., Orčić D. (2022). Comparison Study between Popular Brands of Coffee, Tea and Red Wine Regarding Polyphenols Content and Antioxidant Activity. Food Chem. Adv..

[48] Popov S., Smirnov V., Khramova D., Paderin N., Chistiakova E., Ptashkin D., Vityazev F. (2023). Effect of Hogweed Pectin on Rheological, Mechanical, and Sensory Properties of Apple Pectin Hydrogel. Gels.

[49] El-Sakhawy M., Salama A., Mohamed S.A.A. (2023). Propolis Applications in Food Industries and Packaging. Biomass Convers. Biorefinery.

[50] (2021). Standard Guide for Determination of Thickness of Plastic Film Test Specimens.

[51] (2017). Standard Test Method for Determination of Moisture in Plastics by Loss in Weight.

[52] Kalaycıoğlu Z., Torlak E., Akın-Evingür G., Özen İ., Erim F.B. (2017). Antimicrobial and Physical Properties of Chitosan Films Incorporated with Turmeric Extract. Int. J. Biol. Macromol..

[53] Priyadarshi R., Sauraj, Kumar B., Deeba F., Kulshreshtha A., Negi Y.S. (2018). Chitosan Films Incorporated with Apricot (*Prunus Armeniaca*) Kernel Essential Oil as Active Food Packaging Material. Food Hydrocoll..

[54] (2013). Microbiology of the Food Chain—Horizontal Method for the Enumeration of Microorganisms—Part 1: Colony Count at 30 °C by the Pour Plate Technique.

[55] (2008). Microbiology of Food and Animal Feeding Stuffs—Horizontal Method for the Enumeration of Yeasts and Moulds—Part 2: Colony Count Technique in Products with Water Activity Less than or Equal to 0.95.

[56] (2006). Microbiology of Food and Animal Feeding Stuffs—Horizontal Method for the Enumeration of Coliforms—Colony-Count Technique.

